# ENABLING AGING IN PLACE: THE IMPACT OF HOME MODIFICATIONS ON NURSING HOME ADMISSION RISK

**DOI:** 10.1093/geroni/igad104.3702

**Published:** 2023-12-21

**Authors:** Yuliana Levchenko

**Affiliations:** Portland State University, Portland, Oregon, United States

## Abstract

In an era where the built environment profoundly shapes the ability to age in place, this study aims to investigate the relationship between home modifications and the risk of nursing home admission. Using data from the 2000-2018 Health and Retirement Study, Cox proportional hazard models are employed to analyze the impact of various modifications on admission risk for older adults in the United States (N = 31,133). The home modifications considered include the presence of ramps, railings, wheelchair modifications, grab bars/shower seats, and an emergency call system. The results indicate that having a grab bar/shower seat in one’s home is consistently associated with a decreased risk of nursing home admission (HR = 0.42, CI = 0.34, 0.52). However, there is mixed evidence for the remaining modification. These findings underscore the potential significance of incorporating grab bars and shower seats into building codes and promoting their installation in homes. Such modifications may play a vital role in promoting independent living and reducing reliance on institutional care. Further research is needed to explore the underlying mechanisms by which home modifications influence the risk of nursing home admission.

